# Some Diseases of the Rectum

**Published:** 1884-07

**Authors:** 


### Some of the Diseases of the Rectum, and their Homœopathic and Surgical Treatment. By Mortimer Ayres, M. D. Pp. 78; price, 75 cents. Chicago: Duncan Brothers, 1884.

Under the above title Dr. Ayres treats of rectal abscesses, ulcers, polypi, of hæmorrhoids, fistula in ano, etc. And, curiously enough, he includes constipation as among the diseases of the rectum. Why not include diarrhœa as well? The indications for such remedies as are mentioned for the various diseases are not well stated, and to our mind too many topical applications are advised.

